# Landscape and Future Perspectives of Immunotherapy in Neuroendocrine Neoplasia

**DOI:** 10.3390/cancers12040832

**Published:** 2020-03-30

**Authors:** Ilaria Maggio, Lisa Manuzzi, Giuseppe Lamberti, Angela Dalia Ricci, Nastassja Tober, Davide Campana

**Affiliations:** 1 Department of Experimental, Diagnostic and Specialty Medicine, S. Orsola-Malpighi University Hospital, Alma Mater Studiorum University of Bologna, Via Massarenti 9, 40138 Bologna, Italy; ilaria.maggio@studio.unibo.it (I.M.); lisa.manuzzi@studio.unibo.it (L.M.); angeladalia.ricci@studio.unibo.it (A.D.R.); nastassja.tober@studio.unibo.it (N.T.); 2 NET Team Bologna ENETS Center of Excellence, S. Orsola-Malpighi University Hospital, Alma Mater Studiorum University of Bologna, Via Massarenti 9, 40138 Bologna, Italy; davide.campana@unibo.it; 3 Department of Medical and Surgical Sciences, S. Orsola-Malpighi University Hospital, Alma Mater Studiorum University of Bologna, Via Massarenti 9, 40138 Bologna, Italy

**Keywords:** neuroendocrine tumors, immunotherapy, neuroendocrine neoplasia, neuroendocrine carcinoma, immune checkpoint inhibitors, Merkel cell carcinoma, small cell lung cancer

## Abstract

Background: Neuroendocrine neoplasms are rare entities consisting of a heterogeneous group of tumors that can originate from neuroendocrine cells present in the whole body. Their different behavior, metastatic potential, and prognosis are highly variable, depending on site of origin, grade of differentiation, and proliferative index. The aim of our work is to summarize the current knowledge of immunotherapy in different neuroendocrine neoplasms and its implication in clinical practice. Results: Several studies evaluated the efficacy and safety of immunotherapy in neuroendocrine neoplasms, in any setting of treatment, alone or in combination. Studies led to approval in neuroendocrine neoplasia of the lung, in combination with chemotherapy as first-line treatment or as a single-agent in a third-line setting, and Merkel cell carcinoma as a single agent. Results in other settings have been disappointing so far. Conclusions: Immunotherapy seems a valid treatment option for high grade, poorly differentiated neoplasms. Future trials should explore the combination of immunotherapy with other agents, such as anti-angiogenic or other immunotherapy agents, in order to evaluate potential efficacy in low and intermediate grades, well differentiated tumors.

## 1. Introduction

Neuroendocrine neoplasias (NENs) are rare tumors, but their incidence has increased in the last few years and is estimated to be around 7 cases per 100,000 [1]. NENs are a heterogeneous group of tumors that can originate from neuroendocrine cells present in the whole body. The most frequent origins are the gastroenteropancreatic (GEP) system (around 70% of cases) and lungs (around 20% of cases) [1,2,3]. Less common are NENs originating from the genito-urinary tract, female gynecological system, and Merkel cells in the skin. Their behavior, metastatic potential, and prognosis are highly variable, depending on site of origin, grade of differentiation, and proliferative index. 

GEP–NENs are graded according to the Ki67 proliferation index and grouped into Grades 1 (G1, Ki67 <3%), 2 (G2, Ki67 3–20%), and 3 (G3, Ki67 >20%), following the World Health Organization (WHO) 2010 classification. In this classification, all G3 tumors were referred to as neuroendocrine carcinomas (NECs) and were characterized by aggressive behavior. However, the WHO 2017 classification introduced the category of well-differentiated neuroendocrine tumors (NETs) with Ki67 ≥20% (G3) among pancreatic NENs, with clinical and morphological characteristics halfway between moderately differentiated NETs and NECs [2]. The WHO 2019 classification extended this concept to all gastrointestinal NENs.

Differently, NENs of pulmonary origin are grouped into low-grade (typical carcinoid (TC)), intermediate-grade (atypical carcinoid (AC)), and high-grade (small cell lung cancer (SCLC) and large cell neuroendocrine carcinoma (LCNEC)) [3,4]. This classification reflects the increasing biological aggressiveness and worsening prognosis from TC/AC to SCLC/LCNEC, while TC and AC are considered to have similar behavior, as well as LCNEC and SCLC. NENs show several molecular similarities, irrespective of the site of origin, but different classifications are used depending on the organ of origin [5]. In order to harmonize NEN classification, the International Agency for Research on Cancer (IARC), together with WHO, proposed a new uniform classification system for NENs across different sites. It distinguishes between differentiated NETs and poorly differentiated NECs, supported by morphological, histologic, epidemiologic, genetic, and prognostic differences at specific anatomic sites [6,7].

Merkel cell carcinoma (MCC) is a rare and aggressive NEN of the skin that typically occurs in older patients with a history of sun exposure and can affect patients with an immunodeficiency state. The oncogenic Merkel cell polyomavirus (MCPyV) can be found in 80% of the MCCs [8,9].

The therapeutic approach to NENs is very different and is based on the location of the primary lesion, the morphological differentiation, and the grading. For advanced unresectable low- and intermediate-grade NENs, mainstays of treatment are somatostatin analog (SSA), tyrosine kinase inhibitors (TKIs), mammalian target of rapamycin (mTOR) inhibitors, and peptide receptor radionuclide therapy (PRRT). On the contrary, the backbone of the treatment of advanced high-grade and poorly-differentiated NECs is chemotherapy.

Immunotherapy dramatically changes the natural history of many cancers, and clinical trials are ongoing in different NENs as well. We sought to summarize the current knowledge on immunotherapy in NENs and its implication for clinical practice, highlighting possible new fields of application of this promising therapeutic approach in neglected NENs.

## 2. Materials and Methods

We performed a review of the literature available on Pubmed and of the trials registered on clinicaltrials.gov about immunotherapy with immune checkpoint inhibitors and neuroendocrine neoplasms of any grade and primary site until 28 February 2020. Articles were independently evaluated by two of the authors (I.M. and L.M.) for the relevance to the planned scope of the review.

## 3. Immunotherapy in Human Cancers and Rationale in NENs

**Figure 1:**
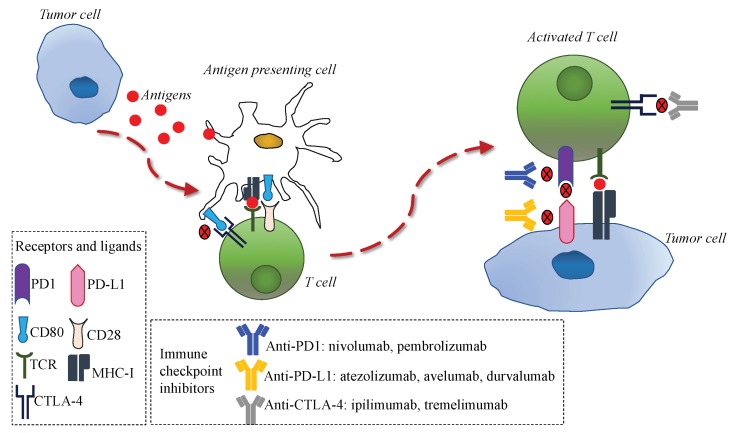
Immune response to tumor cells and main mechanisms of action of anti-programmed cell death 1 (PD-1)/programmed cell death ligand 1 (PD-L1) and anti-cytotoxic T lymphocyte-associated protein-4 (CTLA-4). Tumor cells release antigens that are uptaken by antigen-presenting cells. These cells present tumoral antigens to naïve T-cells, thus activating them. The interaction between PD-1 in activated T-cells and PD-L1 in tumor cells can inhibit the immune response. CD80 on antigen-presenting cells can bind to CTLA-4 on activated T-cells and inhibit the immune response. Anti PD-1/PD-L1 and anti-CTLA4 monoclonal antibodies can bind to PD-1 in activated T-cells, PD-1 in tumor cells, or CTLA-4 in tumor cells, respectively, thus restoring the immune response.

The immune response is directed by the balance between stimulating and inhibitory signals that regulate the action and proliferation of immune cells. The anti-tumor immune response is modulated by the interaction of several proteins located on the membrane of T-cells and antigen-presenting cells (APC), referred to as immune checkpoints [7]. Tumors can escape the immune system recognition through expression ligands that interact with immune checkpoints expressed on T-cells, such as cytotoxic T lymphocyte-associated protein-4 (CTLA-4) and programmed cell death 1 (PD-1) [10,11]. Therefore, in recent years, different types of immune checkpoint inhibitors, consisting of monoclonal antibodies targeting CTLA-4 or PD-1 on T-cell or programmed cell death ligand 1 (PD-L1) on tumor cells, were successfully tested in several tumors (Figure 1) and changed clinical practice, given the improvement in patients’ outcomes [12,13,14,15,16,17,18,19,20,21,22,23,24,25,26,27,28,29,30,31,32,33,34,35,36].

However, there is still limited evidence of the efficacy of immune checkpoint inhibition in many types of NENs, while it proved effective in other difficult-to-treat NENs, such as SCLC and MCC [20,22,23,37].

## 4. Predictive Biomarkers for Immunotherapy

As the therapeutic scenario for NENs changes with the addition of immunotherapy, so does the need to find predictive biomarkers that can guide clinical decisions. Since benefit on immunotherapy treatments is usually limited to a subset of patients, great effort has been made by the research community to find predictive factors able to identify such patients. Studies in commonly immunotherapy-treated tumors, such as malignant melanoma and non-small cell lung cancer, have identified biomarkers that might have the potential to predict response to immunotherapy. In particular, high levels of PD-L1 expression and a high tumor mutational burden (TMB), defined as the number of non-synonymous mutations per megabase of sequenced genome, were associated with an increased benefit to immunotherapy [38,39,40]. However, durable responses to immunotherapy also occur in patients whose tumors have low or no PD-L1 expression and low TMB so that using only these two biomarkers could exclude potential responders from treatment [39,40].

While the higher PD-L1 expression is intuitively linked to the mechanism of action of immune checkpoint inhibitors, the role of TMB is still under debate. It is likely that a higher number of somatic mutations increase the likelihood of generating neoantigens that can be immunogenic and recognized by T-cells to trigger an immune response [40].

With regard to neuroendocrine tumors, most evidence comes from SCLC. The rationale for the use of immunotherapy in this setting derives from the fact that SCLC has one of the highest TMB among human cancers (median 8 mutations per megabase (mut/Mb)), despite expressing PD-L1 in only 20% of the cases [41,42,43]. However, TMB alone does not completely predict benefit from immune checkpoint inhibition and should be integrated with other pathological and genetic factors in additional models to improve biomarker performances [44].

There are few studies supporting the use of immunotherapy in GEP–NENs. Expression of PD-L1 and PD-1 is associated with higher-grade tumors (i.e., NET G3 and NECs) and with worse progression-free survival (PFS) and overall survival (OS) [45,46,47,48]. A recent work on NETs of different grades and primary sites, including pancreas, midgut and lung, analyzed expression characteristics of PD-L1, PD-L2, indoleamine-deoxygenase-1 (IDO-1), tumor-infiltrating T-lymphocytes (TILs), as well as biomarkers of hypoxia and angiogenesis [49]. Among 102 NET samples, PD-L1 expression was highest in lung NETs, and lowest in ileal NETs, while PD-L2 expression was highest in pancreatic NETs. Furthermore, exhausted and regulatory TILs were enriched in PD-L1-positive NETs but decreased in G3 well-differentiated NETs. This suggests that immune tolerance in NETs might be driven by PD-L1/2 expression and that NETs that express PD-L1 and with TILs might benefit from PD-L1 inhibition. Microsatellite instability (MSI) is a tumor-agnostic marker of response to PD-1/PD-L1 blockade [29] and can be found in GEP mixed adeno-neuroendocrine carcinoma (MANEC), while it is rare in G1-G2 GEP-NET [50,51]. Furthermore, TMB is typically low in G1–G2 NENs, while it is higher in NECs, as reported in a study that used 17 mut/Mb as a cut-off to define high and low TMB tumors [52,53].

Given the urgent need for identifying reliable predictive factors, several ongoing clinical trials include a biomarker analysis to identify factors able to predict for response to immunotherapy (e.g., NCT03095274, NCT03074513, NCT03728361). The latter trial (NCT03728361) investigates the efficacy of the combination of nivolumab and temozolomide, an alkylating agent, in NET of any grade, NEC, and SCLC. Among the exploratory objectives of this study, there are to be determined whether baseline TMB is predictive for response to therapy in SCLC patients, whether changes in blood-based TMB during treatment may predict clinical benefit in the whole population, and whether a composite immune- and tumor-cell-staining score can be developed, with or without PD-L1 by immunohistochemistry, to predict response in the SCLC cohort.

## 5. Immunotherapy in Lung NENs

SCLC is the most common NEN and has been the most investigated NEN in immunotherapy trials to date (Table 1).

Around 70% of SCLC patients are diagnosed at the extensive-stage of the disease and have a dismal prognosis, with a median overall survival (OS) of 10 to 12 months [67]. For many years, the standard first-line treatment was chemotherapy with platinum and etoposide, while topotecan is currently the only approved treatment in the second-line setting [67].

Systemic therapy for SCLC had not changed in decades until the recent introduction of immunotherapy.

### 5.1. SCLC First Line and Maintenance 

The therapeutic approach of SCLC is currently changing based on the result of two randomized controlled trials. The IMpower-133 compared carboplatin-etoposide plus atezolizumab, an IgG4 Fc modified anti-PD-L1 humanized antibody, or placebo for four cycles followed by atezolizumab or placebo as maintenance until disease progression or unacceptable toxicity [22]. Coprimary endpoints in this study were PFS and OS. The study showed an improved PFS (median PFS (mPFS) 5.2 vs. 4.3 months in the atezolizumab vs. placebo arm, respectively; hazard ratio, HR 0.77; 95% CI: 0.62–0.96; *p* = 0.02) and OS (median OS (mOS) 12.3 vs. 10.3 months; HR 0.70; 95% CI: 0.54–0.91; *p* = 0.007). Overall response rate (ORR) was 60.2% vs. 64.4% (HR 1,56; 95% CI: 1.10–2.22) and median duration of response (mDOR) was 4.2 (1.4–19.5) vs. 3.9 (2.0–16.1) months in the atezolizumab vs. placebo arm, respectively. Immune-related adverse events (irAEs) were reported in 39.9% of patients in the atezolizumab arm, compared to 24.5% in the placebo arm, and the most common were rash (any grade 18.7%) and hypothyroidism (any grade 12.6%). Although the benefit was minimal and is likely limited to a subset of patients, which, to date, cannot be identified [68], this is the first strategy to improve ES-SCLC survival in decades, so that the combination of chemotherapy and atezolizumab has been approved by the Food and Drug Administration (FDA) in first-line treatment of SCLC.

In the phase III CASPIAN trial, patients with untreated SCLC were assigned to received durvalumab, an IgG1 kappa anti-PD-L1 monoclonal human antibody, plus platinum-etoposide or durvalumab plus tremelimumab (an IgG2 anti-CTLA-4 fully human monoclonal antibody) plus platinum-etoposide followed by durvalumab as maintenance, until disease progression or unacceptable toxicity, or chemotherapy alone [23]. The primary endpoint was overall survival in the intention-to-treat population. Results of the tremelimumab plus durvalumab arm are not available yet, but results of the durvalumab plus chemotherapy and chemotherapy alone arms have been published. The combination of durvalumab plus chemotherapy yielded longer OS than chemotherapy alone (mOS 13.0 vs. 10.3 months, respectively; HR 0.73; 95% CI: 0.59–0.91; *p* = 0.0047). The median PFS, which was a secondary endpoint, was 5.1 vs. 5.4 months (HR 0.78; 95% CI: 0.65–0.94), in the durvalumab plus chemotherapy and chemotherapy alone arms, respectively. ORR was 68% vs. 58%, and mDOR was 5.1 (95% CI: 3.4–10.4) vs. 5.1 months (95% CI: 3.7–6.8) in the durvalumab arm compared with the chemotherapy arm, respectively. Overall, irAEs occurred in 20% and 3% of patients in the durvalumab and control arms, respectively, the most common being hypothyroidism and hyperthyroidism (in 9% and 5% of patients, respectively). Grade 3 or 4 irAEs occurred in 5% of patients in the durvalumab arm and 1% of patients in the control arm.

By contrast, a phase III randomized study evaluating the efficacy of ipilimumab (an anti-CTLA-4 fully human monoclonal antibody) added to platinum-based chemotherapy vs. chemotherapy and placebo failed to meet its primary endpoint of showing an increased OS (mOS 11 vs. 10.9 months). The mPFS was 4.6 months in the ipilimumab arm vs. 4.4 months in the placebo arm, while ORR was 62% in both arms, for mDOR of 4.0 (95% CI: 3.32–4.17) vs. 3.5 months (95% CI: 3.25–4.07) in the ipilimumab and placebo arms, respectively. Treatment-related serious adverse events diarrhea (8%) and colitis (5%) were more common in the ipilimumab than in the placebo arm (1% each) [54]. Furthermore, the phase III trial CheckMate-451, enrolling 834 patients whose disease had not progressed after four cycles of platinum-based chemotherapy, tested the efficacy of nivolumab, an IgG4 anti-PD1 fully human monoclonal antibody, plus ipilimumab every three weeks for four cycles followed by nivolumab or nivolumab alone every two weeks or placebo as maintenance treatment. Treatments were administered until progression or unacceptable toxicity. The primary endpoint of the study was OS. However, the study failed to show a survival improvement, with mOS of 10.4 vs. 9.2 vs. 9.6 months in the nivolumab, nivolumab plus ipilimumab, and placebo arms, respectively. The mPFS was 1.9 vs. 1.7 vs. 1.4 months in the nivolumab, nivolumab plus ipilimumab, and placebo arms, respectively. However, in the nivolumab plus ipilimumab arm, there was a higher rate of Grade 3–4 adverse events (52%), of discontinuations due to toxicity (31%), and of treatment-related deaths (2.5%) compared to the other arms [55].

### 5.2. SCLC Second and Third Line

Trials comparing single-agent immune checkpoint inhibitors with standard second-line chemotherapy (i.e., topotecan and amrubicin) failed to prove their superiority in an unselected SCLC population. The phase III randomized CheckMate-331 trial, comparing nivolumab vs. topotecan or amrubicin as second-line treatment after a first-line platinum-based regimen, administered until progression or unacceptable toxicity, did not show an improvement in OS for the experimental vs. the control arms, with mOS of 7.5 vs. 8.4 months, respectively (HR 0.86 (95% CI: 0.72–1.04)). The mPFS was 1.5 vs. 3.8 months (HR 1.41 (95% CI: 1.18–1.69)) while ORR was 14% vs. 16% with an mDOR 8.3 vs. 4.5 months, in the nivolumab and the topotecan arms, respectively. However, nivolumab was better tolerated than topotecan (all grade AEs: 55% vs. 90%; Grade 3–4 AEs: 14% vs. 73%, respectively) [56]. A similar phase II trial of atezolizumab as second-line treatment after first-line with platinum-based chemotherapy vs. topotecan or re-challenge with carboplatin and etoposide did not meet its primary endpoint of ORR (2.3% in the atezolizumab arm vs. 10% in the chemotherapy arm), while the mPFS was 1.4 months with atezolizumab and 4.3 months with topotecan. Atezolizumab was well tolerated and only a 4.2% of patients experienced Grade 3 fatigue and two others Grade 1 thyroid function alteration [59].

In the phase I-II CheckMate-032 trial, SCLC patients who had been treated at least with one prior platinum-containing chemotherapy, were assigned to receive nivolumab 3 mg/kg every two weeks or nivolumab 1 mg/kg plus ipilimumab 1 mg/kg or nivolumab 1 mg/kg plus ipilimumab 3 mg/kg or nivolumab 3 mg/kg plus ipilimumab 1 mg/ every three weeks for four cycles, followed by nivolumab 3 mg/kg every two weeks until disease progression or unacceptable toxicity [57]. The majority of patients had three or less previous lines of treatment with three or more previous treatments received by 3% to 10% of patients across different arms. The primary endpoint was ORR, which was 10% in the arm with nivolumab single agent, 23% in the arm receiving nivolumab 1 mg/kg plus ipilimumab 3 mg/kg, and 19% in patients receiving nivolumab 3 mg/kg plus ipilimumab 1 mg/kg. One-year OS was 33% in the arm with nivolumab single agent, 43% for the nivolumab 1 mg/kg plus ipilimumab 3 mg/kg cohort, and 35% in patients receiving nivolumab 3 mg/kg plus ipilimumab 1 mg/kg. The mDOR was not reached (95% CI: 4.4—not reached) with nivolumab 3 mg/kg, 7.7 months (4.0—not reached) with nivolumab 1 mg/kg plus ipilimumab 3 mg/kg and 4.4 months (3.7—not reached) with nivolumab 3 mg/kg plus ipilimumab 1mg/kg. The time to objective response was 2.0 months (1.3–2.8) in the nivolumab 3 mg/kg arm, 2.1 months (1.4–2.8) in the nivolumab 1 mg/kg plus ipilimumab 3 mg/kg arm, and 1.4 months (1.3–2.7) in the nivolumab 3 mg/kg plus ipilimumab 1 mg/kg arm. Grade 3–4 treatment-related adverse events (TRAEs) occurred in 13% of patients in the nivolumab 3 mg/kg cohort, 30% of patients in the nivolumab 1 mg/kg plus ipilimumab 3 mg/kg cohort, 19% in the nivolumab 3 mg/kg plus ipilimumab 1 mg/kg cohort. There were no Grade 3-4 AEs in the nivolumab single-agent cohort. The most common Grade 3–4 AEs were increased serum lipase levels and diarrhea. A subsequent analysis, evaluating the efficacy of nivolumab in SCLC patients who received nivolumab monotherapy as a third-line treatment, showed an ORR of 11.9%, with a median DOR of 17.9 months (range 3.0–42.1 months) [69]. Based on these results, the FDA approved nivolumab monotherapy in the third-line treatment of SCLC.

The phase II KEYNOTE-158 study evaluated the efficacy of the IgG4 kappa anti-PD1 humanized antibody pembrolizumab in 11 different types of solid tumors. Among them, 107 pre-treated patients with SCLC (79% had 1–2 prior lines of chemotherapy) were treated for two years or until progression or intolerable toxicity. Of these, 42 (39%) had SCLC with PD-L1 expression ≥1%. The primary endpoint was ORR, which was 35.7% in the PD-L1-positive group and 6% in the PD-L1 negative group. The mPFS was 2.1 months in the PD-L1-positive group and 1.9 months in the PD-L1-negative group. The mOS was 14.6 months in the PD-L1-positive group and 7.7 months in the PD-L1-negative group [58]. The phase Ib trial KEYNOTE-028 enrolled 24 patients with PD-L1 positive (≥1%) SCLC to receive pembrolizumab for two years or until progression or unacceptable toxicity. No prior treatment was received by 8.4% of patients, 46.5% received 1-2 prior lines of chemotherapy, 26.5% of patients received 3–4 prior lines, and 8.8% received 5 or more lines of treatment. ORR, the primary endpoint, was 33%. The mPFS was 1.9 and the mOS was 9.7 months [61]. A pooled analysis of 83 patients from the SCLC cohorts of both KEYNOTE-158 and KEYNOTE-028 trials who had received two or more prior lines of therapy showed an ORR of 19%, with complete response in 2% of patients and stable disease in 18%. The mDOR was not reached, and 9 of 16 responders had a response lasting more than 18 months. The mPFS was 2.0 months, and the mOS was 7.7 months. Irrespective of prior therapies, 8% of patients had Grade 3 TRAEs, and three patients had Grade 5 adverse events (intestinal ischemia, pneumonia, encephalopathy) [70]. Based on efficacy data from the pooled analysis, FDA has approved pembrolizumab for patients with disease progression to two previous lines of treatment, including platinum-based chemotherapy.

Another phase II trial of the combination of paclitaxel every three weeks for six cycles plus pembrolizumab from the second cycle until disease progression or unacceptable toxicity as second-line treatment after platinum-etoposide chemotherapy showed a moderate activity, with a 23.1% of ORR with an mDOR of 9.1 months, an mPFS of 5.0 months, and mOS of 9.1 months. The most common Grade 3–4 adverse events were febrile neutropenia (7.7%), asthenia (7.7%), hyponatremia (7.7%), and type I diabetes (7.7%) [60].

### 5.3. LCNEC, Typical and Atypical Carcinoid

Usually, LCNEC patients are excluded from studies on SCLC, so data about immune checkpoint inhibition efficacy are derived mainly from case reports [71,72].

Recently, six French centers have retrospectively analyzed 10 patients with advanced LCNEC treated with nivolumab or pembrolizumab after platinum-based first-line therapy. The study showed a partial response in 60% and a stable disease in 10% of patients. The mPFS was 57 weeks. These data seem to demonstrate a promising activity of immunotherapy in this setting [73].

In respect to TC and AC, the prospective evidence available comes from the phase Ib KEYNOTE-028 study, in which 25 patients with TC or AC were enrolled. The primary endpoint was ORR, which was 12%, while 15 patients had stable disease. The 12 months-PFS rate and OS rate were 27% and 65%, respectively [61]. A recent phase II trial evaluated the efficacy of spartalizumab (PDR001), an anti-PD-1 humanized IgG4 monoclonal antibody, in patients with advanced well-differentiated neuroendocrine tumors of pancreatic (N = 30), gastrointestinal (N = 30) or thoracic origin (N = 30) after progression to at least one prior therapy, including everolimus, or poorly-differentiated gastroenteropancreatic neuroendocrine carcinoma (GEP–NEC, N = 20), who have progressed on one line of chemotherapy, regardless of PD-L1 expression. The primary end point was an ORR of at least 20% in the cohort of well-differentiated NET. The ORR was overall 7.4%, but it reached 20% in the thoracic NET cohort, suggesting some signal of activity in this setting. The most common Grade 3-4 adverse events were abdominal pain, anemia, dyspnea, and hypertension [62].

The DART study is a currently ongoing phase II basket trial of double blockade with nivolumab 240 mg every 2 weeks plus ipilimumab 1mg/kg every 6 weeks on a continuous schedule until disease progression or unacceptable toxicity, across multiple rare tumor types. Recently, data from the neuroendocrine cohort (excluding pNET) were reported. Of the 32 patients enrolled in this cohort, 18 had high-grade neuroendocrine carcinoma. The most common primary sites were gastrointestinal (N = 15) and lung (N = 6); median number of prior lines of therapy was 2. The primary end point was ORR, which was 25% in the entire cohort, but up to 44% (8/18) in high-grade neuroendocrine carcinoma vs. 0% in low/intermediate grade tumors. The mPFS was 4 months and the mOS was 11 months. irAEs were reported in 72% of patients: the most common were hypothyroidism (31%) and AST increase (25%), while the most common Grade 3–4 irAEs (38%) were ALT increase (9%) and AST increase (6%), lipase increase (6%), and encephalopathy (6%) [64].

## 6. Immunotherapy in Merkel Cell Carcinoma

Immunotherapy has revolutionized the therapeutic approach and prognosis of MCC [74]. MCC is characterized by a high recurrence rate even after surgical resection and adjuvant radiotherapy of locoregional disease [75]. Even Stage I–II disease after radical treatment have a recurrence rate of 35% at three years [76]. For many years, the only therapeutic option for advanced disease has been platinum agents and etoposide chemotherapy. MCC is often initially responsive to chemotherapy, but the response is not durable, with poor prognosis upon relapse/progression and a mortality rate of 33% [77,78].

There is compelling evidence about the role of the immune system in MCC development and prognosis definition. Indeed, MCC is more common in patients with a chronic immunosuppressive state, like acquired immunodeficiency syndrome from HIV infection, solid organ transplant, or B-cell malignancies, accounting for about 8% of the MCC cases [78]. Lymphocyte infiltration is a strong predictor of disease outcome, and CD8+ lymphocyte gene expression is associated with a better prognosis in terms of survival [79,80]. In a subset of patients, primary MCC cannot be identified, since the regression of primary lesion can occur. These patients have a better prognosis than those with an identified primary MCC lesion, suggesting that immune response can help achieve better tumor control [81,82,83,84].

MCPyV is integrated into MCC tumor DNA in 80% of cases and induces the expression of non-self viral antigens on the surface of tumor cells that can be recognized by adaptive and innate immune response cells. The “large T-antigen” produced upon MCPyV integration inactivates the tumor suppressors retinoblastoma 1 (Rb1) and p53, which is functional to viral spread and triggers oncogenesis in MCC [85,86]. Virus-negative MCC can occur and usually derives from ultraviolet light exposure, which determines the accumulation of somatic DNA mutations (increasing TMB) and a consequent high probability for neoantigens formation that increases tumor immunogenicity [87].

As in other highly immunogenic tumors such as NSCLC and malignant melanoma, PD-L1 is expressed by both virus-positive and virus-negative MCC, in both tumor cells and infiltrating immune cells [86]. Currently, there are different immune checkpoint inhibitors approved for advanced MCC in first and further lines of therapy (Table 1).

Pembrolizumab 2 mg/kg every 3 weeks for up to 2 years was evaluated in a phase II, single-arm, multicenter trial in previously untreated patients with advanced MCC. The primary endpoint was ORR. Pembrolizumab was associated with an ORR of 56% (59% in virus-positive and 53% in virus-negative), independently from PD-L1 expression [20]. The 24-month PFS rate was 48.3%, and the mPFS time was 16.8 months, while the 24-month OS rate was 68.7%, and mOS time was not reached. Among 28 responders, the median time to response was 2.8 months (range: 1.5 to 9.7), and mDOR was not reached (range: 5.9 to 34.5+). TRAEs events of any grade and Grades 3–4 were reported in 96% and 28% of patients, respectively. Seven patients (14%) discontinued pembrolizumab because of adverse events, and there was one death attributed to treatment. Hypothyroidism (6%), pneumonitis (6%), pancreatitis (4%), and maculopapular rash (4%) were the most common irAEs.

Avelumab, an IgG1 anti-PD-L1 human antibody, is being tested in the ongoing phase III Javelin Merkel 200 trial as first-line treatment in MCC. Avelumab is administered at 10 mg/kg dose every 2 weeks until confirmed disease progression, unacceptable toxic effects, or consent withdrawal. No previous treatment for metastatic disease was allowed, but 7.7% and 5.1% of patients had received previous chemotherapy as adjuvant treatment and for locally advanced disease, respectively. A preplanned interim analysis carried on in 29 patients showed an ORR (the primary endpoint) of 62.1%, with a duration of response of at least 6 months in 83% of responding patients [66]. Any grade and Grade 3 TRAEs were reported in 71.8% and 20.5% of patients, respectively, while Grade 1 irAEs occurred in 15.4% of patients.

Avelumab has been evaluated also in pretreated advanced MCC in an open-label, single-arm, multicenter, phase II trial [21]. Patients progressed to at least one previous line of chemotherapy, and in particular, 59%, 30%, 8%, 3% of patients received one, two, three, or four previous lines of chemotherapy, respectively. The updated results at a median follow up of 16.4 months showed that avelumab provided for an ORR, the primary endpoint of the study, of 33%, with an estimated 74% of responses that lasted more than 1 year and an mDOR that was not reached (95% CI: 18.0—not reached). One-year PFS and OS rates were 30% and 52%, respectively, with an mOS of 12.9 months. Moreover, responses were independent of the presence of MCPyV and PD-L1 expression, but subgroup analyses suggested a higher probability of response in patients receiving fewer prior lines of chemotherapy and with PD-L1 positive tumors [37]. TRAEs occurred in 70% of patients, and Grade 3 was reported in 5% of cases. TRAEs occurring in more than 10% of patients were fatigue (24%) and infusion-related reactions (17%).

Another immune checkpoint inhibitor investigated in advanced MCC is nivolumab. A cohort of the phase I/II CheckMate-358 study included 25 MCC patients, either treatment-naïve or previously treated with two or less lines of therapy. Nivolumab 240 mg was administered every two weeks until progression or unacceptable toxicities. At a median follow up of 51 weeks, ORR was 64%, irrespective of PD-L1 expression and MCPyV status, but it was higher in treatment-naïve patients (71%) than in previously treated ones (63%) [88]. At three months, PFS and OS rates were 82% and 92%, respectively. Among the 22 patients evaluable for response, the median time to response was 2.0 months (range 1.8–5.3) and mDOR was not reached (range 0.0–5.6). TRAEs of any grade and Grades 3–4 were reported in 68% and 20% of patients, and 12% of patients discontinued nivolumab because of toxicity.

This data suggests that immunotherapy might be more effective in treatment-naïve patients than in those previously treated, but these findings require validation in larger patient cohorts [81].

On the basis of these results, immune checkpoint inhibition with PD-1/PD-L1 blockade is approved as first-line or subsequent-line treatment of MCC by the FDA (avelumab, pembrolizumab) and the European Medicine Agency (avelumab).

## 7. Immunotherapy in Gastroenteropancreatic NENs

The current knowledge about immunotherapy in GEP-NENs is mainly based on a phase Ib study and some case reports. In the phase Ib KEYNOTE-028 study, 16 patients with pancreatic NEC received pembrolizumab monotherapy, showing an ORR of 6% and 12 months-PFS and OS rates of 27% and 87%, respectively [61].

Cases of activity of PD-1/PD-L1 immune checkpoint inhibition are mainly reported in NECs [89,90,91]. The phase II spartalizumab trial also includes a cohort of patients with well-differentiated gastrointestinal (N = 30) and pancreatic NET (N = 30) and a cohort of patients with poorly differentiated GEP-NEC (N = 20). In these cohorts, ORR was 0%, 3.0%, and 4.8%, respectively, showing a marginal activity in this setting [62].

Recently, toripalimab, a humanized IgG4 antibody anti PD-1 receptor, was tested in a phase Ib trial in pre-treated NEN patients (Ki67 >10%) for up to 24 months or until disease progression or intolerable toxicity. Eight patients had a well-differentiated NET (WD-NET), and 32 patients, a poorly differentiated NEC (PD–NEC). Sixty-five percent of the patients had received one previous line of therapy, 17.5% two previous lines of treatment, and 17.5% more than three lines. Nine patients presented a pancreatic origin, while, among extra-pancreatic origin, the most common were colorectal, stomach, duodenum, and esophageal primary. ORR was 20%, and median DOR was 15.2 months. ORR was higher in patients with PD-L1 expression ≥10% (50.0% vs. 10.7%, *p* = 0.019) or high TMB (75.0% vs. 16.1%, *p* = 0.03). Interestingly, 3/8 responders had an *ARID1A*-mutant tumor, while the same mutation was present in only 1/27 of the non-responder tumors. According to morphology, ORR was 18.7% in the PD–NEC subgroup and 25% in the WD–NET subgroup. Also, improved mPFS and mOS were observed in patients with PD-L1 ≥10% (mPFS 3.8 vs. 2.2 months, *p* = 0.07 and mOS 9.1 vs. 7.2 months, *p* = 0.15). Grade 3 AEs were reported in 25% of patients, including increased lipase, hyperglycemia, increased bilirubin, aspartate aminotransferase, creatine kinase, elevated lactate dehydrogenase and thrombocytopenia; one patient presented Grade 4 increased alanine transaminase. irAEs were observed in 55% of patients, the most common being transaminase increase, elevated bilirubin, and hypothyroidism [63].

In the DART trial (NCT02834013), 15 patients with GI NET (without pNET) received nivolumab plus ipilimumab: objective responses were observed only in high-grade NEC (ORR: 44%), with poor efficacy in the well-differentiated forms [64].

A phase II basket trial of atezolizumab 1200 mg and bevacizumab 15mg/kg every 3 weeks in solid tumors also included NEC and NET patients (NCT03074513). Recently, data from the pancreatic and extra-pancreatic NET cohorts (extra-pNET), each enrolling 20 pre-treated patients with Grades 1–2 NET, were presented and showed good clinical activity. ORR, the primary endpoint, was 20% and 15%, and the mPFS was 19.6 months (95% CI: 10.6—NR) and 14.9 months (95% CI: 6.1—NR) in the pNET cohort and in the extra-pNET cohort, respectively [65].

The current knowledge of the efficacy of immunotherapy in GEP–NENs is still not mature and, even if some evidence suggests the potential for good outcomes with immune checkpoint inhibitors, more prospective robust data are needed to assess the real value of this therapeutic approach in this subset of neuroendocrine neoplasms. Nevertheless, the low TMB and usually “cold” immune microenvironment suggest that combination therapies might be used to overcome NEN intrinsic resistance to immunotherapy.

Immunotherapy in unselected GEP–NENs seems unfeasible since a small fraction of patients derived benefit from it. However, identification of predictive biomarkers, such as high TMB and *ARID1A* mutations, should be investigated to identify best GEP–NENs patients to offer immunotherapy to.

## 8. Ongoing Clinical Trials and Future Perspectives

Ongoing trials (summarized in Table 2) and future efforts should focus on remodeling the host immune system and the tumor microenvironment in order to develop a more effective treatment strategy and to enhance the effect of immune checkpoint inhibitors in those tumors that seems to have a low immunogenic state.

### 8.1. Pulmonary High-Grade NENs

While studies of immune checkpoint inhibitors in monotherapy have often proven inferior to standard chemotherapy, the most recent studies show that a combination strategy is more effective in leading to a survival advantage in SCLC [22,23]. Several trials are ongoing in SCLC investigating immunotherapy in combination either with chemotherapy or with other immune checkpoint inhibitors, in order to achieve a synergistic effect. In addition, new drugs targeting different components of the immune system are currently under evaluation in combination with anti-CTLA-4 or anti-PD-1/PD-L1 to enhance the immune response against tumor cells. 

Utomilumab is an agonistic monoclonal antibody targeting CD137, a co-stimulatory receptor expressed on several immune cells, like T-cells and NK cells. The phase Ib/II multicenter JAVELIN Medley trial of utomilumab plus avelumab in several metastatic solid tumors, including SCLC, is currently ongoing (NCT02554812).

INCAGN01876 is a new experimental drug that binds to glucocorticoid-induced TNF-receptor-related protein (GITR) as a co-stimulatory agent for T-cell receptors. INCAGN01876 is being evaluated in combination with ipilimumab and nivolumab in a phase I/II study that also recruits patients with SCLC (NCT03126110). A phase I/II trial of INCAGN01949, an agonistic drug targeting CD134, another T-cell costimulatory receptor, in combination with nivolumab or ipilimumab or both is actually completed and pending publication of results (NCT03241173).

Poli-ADP-ribose-polymerase (PARP) inhibitors are approved in ovarian and breast cancers in patients harboring *BRCA* gene mutations. Targeting DNA damage response through PARP inhibition leads to the upregulation of PD-L1 expression on SCLC cells and potentially enhances the activity of anti-PD-L1 immunotherapy [92]. The MEDIOLA trial is a phase I/II basket trial of durvalumab in combination with olaparib (a PARP inhibitor), in advanced solid tumors, whose primary endpoint is ORR [93]. Twenty relapsed SCLC patients received olaparib monotherapy for 4 weeks, then olaparib plus durvalumab until disease progression. ORR was 11% while mPFS was 3.0 months, and mOS was 8.8 months. A similar phase II trial of rucaparib, another PARP inhibitor, in addition to nivolumab in platinum-sensitive SCLC patients as maintenance after induction therapy with platinum doublet chemotherapy, is ongoing (NCT03958045).

The Notch signaling pathway is involved in many processes like proliferation and differentiation towards a neuroendocrine phenotype. Notch receptors can induce neuroendocrine differentiation through binding to its ligands (Delta-like1 (DLL1), Delta-like3 (DLL3), and Delta-like4 (DLL4)), and interact with other signaling pathways like PI3K/Akt/mTOR, NFkB, and APC/beta-catenin, resulting in oncogenic and tumor-suppressive signaling, which is often present in neuroendocrine tumors [94]. Based on this evidence, antibody-drug conjugates targeting DDL3, one of the Notch receptor family ligands, were developed. Rovalpituzumab Tesirine (Rova-T) was tested as a single agent in the phase III MERU trial as maintenance therapy following first-line, platinum-based chemotherapy in 740 advanced SCLC patients. A pre-planned interim analysis, however, demonstrated a lack of survival improvement compared with placebo [95]. In the phase II TRINITY trial, 339 SCLC patients pre-treated with at least two prior lines of therapy received Rova-T once every 6 weeks for two cycles. The primary endpoints were ORR and OS. The observed activity was modest, ORR was 12.4% in all populations, and 14.3% in DLL3-high patients, while mOS was 5.6 months in all patients and 5.7 months in DLL3-high patients [96]. Since Rova-T offered disappointing results as monotherapy, it was tested in a phase I/II study in combination with nivolumab or nivolumab plus ipilimumab after at least one line of platinum-based chemotherapy (NCT03026166). In some patients, durable responses were observed: mDOR was 3.8 months (95% CI: 1.6–5.2) in the nivolumab/ipilimumab arm and 3.3 months (95% CI: 1.4–5.7) in nivolumab arm. The toxicity profile of the association of Rova-T with nivolumab–ipilimumab was unfavorable, while Rova-T plus nivolumab demonstrated a better safety profile [97]. Plinabulin, an inhibitor of tubulin polymerization, is currently being tested in a phase I/II trial, in association with nivolumab plus ipilimumab (ipilimumab for four cycles) in relapsed SCLC patients who progressed after at least one platinum-based chemotherapy regimen (NCT03575793).

Cancer vaccines aim to elicit an immune response against tumors, exploiting different kinds of antigens, but the immune response can be hampered by tumor immune escape through immune checkpoint expression. This is the rationale of combining vaccines and immune checkpoint inhibition, as tested in many cancers, including MCC (NCT04160065). A phase II trial of the combination of nivolumab and ipilimumab plus a dendritic cell-based p53 vaccine (Ad.p53-DC) in relapsed SCLC patients after at least one prior treatment with a platinum based-regimen, is currently ongoing (NCT03406715).

Resistance to immune checkpoint inhibition may be driven by multiple mechanisms, but the immunosuppressive role of the vascular endothelial growth factor (VEGF) is gaining increasing interest so that combined VEGF and immune checkpoint inhibition is being tested in different cancer types, and proved to be effective in kidney cancer [98,99]. Following the promising results of the phase II trial of single-agent anlotinib [100], a phase II/III trial of anlotinib, a multikinase inhibitor targeting VEGF receptor, fibroblast growth factor receptor (FGFR), platelet-derived growth factor receptors (PDGFR), and c-Kit plus sintilimab, a human IgG4 monoclonal antibody against PD-1, in relapsed SCLC patients after first-line platinum-etoposide chemotherapy is ongoing [NCT04192682].

Compared to SCLC, there are only a few prospective trials specifically designed for the combination of immunotherapy and chemotherapy in LCNEC. Two phase II trials of pembrolizumab in combination with platinum (carboplatin or cisplatin) and etoposide chemotherapy (NCT03901378) and of nivolumab plus ipilimumab (NCT03591731) in metastatic or unresectable relapsed LCNEC or GEP–NEC in progression after one or two lines of treatment, including a platinum-based regimen, are ongoing.

Similar to the anlotinib plus sintilimab combination in SCLC, the combination of VEGF and immune checkpoint inhibition is being tested in a phase II trial of cabozantinb, a multikinase inhibitor also targeting VEGF and c-MET, combined with nivolumab and ipilimumab in patients with poorly differentiated NEN (including small and large cell morphology), regardless of the primary site, progressed on one previous line of treatment (NCT04079712). The combination therapy of nivolumab with temozolomide is being tested in patients with recurrent or refractory SCLC, LCNEC, or advanced G1-G3 well-differentiated NETs (NCT03728361).

### 8.2. Well-Differentiated GEP- and Lung NENs

The phase II DUNE trial of the combination of durvalumab and tremelimumab is ongoing in G1/G2 neuroendocrine tumors of the pancreas, gastrointestinal tract, and lung and G3 of the GEP system or unknown primary site (excluding lung primaries) in pre-treated patients. The primary endpoint is the clinical benefit rate, defined as the percentage of patients achieving complete response, partial response, or stable disease at the ninth month (NCT03095274). In the phase II NCT03074513 trial, bevacizumab and atezolizumab are tested in solid tumors, including NECs and NETs of any site. Primary endpoint is ORR, and the identification of predictive and prognostic biomarkers is an explorative objective. While some preliminary results from the NETs cohort have already been presented, results from the NEC cohort are eagerly awaited.

### 8.3. Merkel Cell Carcinoma

After the approval of immunotherapy for first and subsequent line treatment in MCC, new trials are focusing on moving immunotherapy in the adjuvant setting in Stage I–III MCC. Trials of nivolumab (NCT02196961), avelumab (NCT03271372 and NCT04291885), and of pembrolizumab (NCT03712605) in the adjuvant setting are ongoing. Safety and tolerability of nivolumab with radiation therapy or ipilimumab as adjuvant therapy are being tested in a phase I trial as well (NCT03798639).

Another interesting field of research is the association of immune checkpoint inhibitors and radiation therapy. The combination of immune checkpoint inhibitors with radiation therapy can create synergistic anti-tumor activity, eliciting a stronger immune response. The rationale for the use of radiotherapy lies in the so-called “abscopal effect”. This is an immune-mediated effect triggered by a localized radiation treatment at one site that recruits reactive cells that elicit a response on a distant site outside of the irradiated field [101,102,103,104]. A phase I/II trial is testing the combination of localized radiotherapy or recombinant interferon-beta (that is a known immune stimulator) and avelumab, with or without cellular adoptive immunotherapy in advanced MCC (NCT02584829). Furthermore, two phase II randomized trials are currently evaluating the combination of nivolumab and ipilimumab (NCT03071406) and pembrolizumab (NCT03304639), each with or without stereotactic body radiotherapy for metastatic MCC. Pembrolizumab combined with radiation therapy is also being tested in a single-arm phase II trial (NCT03988647) in MCC patients who have received up to 3 treatments, including adjuvant treatment, so that untreated patients are also eligible. 177-Lu-DOTATATE is a peptide receptor radionuclide therapy that uses somatostatin analogs marked with a radioactive isotope (177-Lutetium) to target and kill neuroendocrine tumor cells that express somatostatin receptors and significantly improve outcomes in somatostatin receptor-expressing NET progressing after first-line somatostatin analog treatment [105]. Avelumab will be tested in combination with external beam radiation therapy or 177-Lu-DOTATATE in an interesting phase I/II trial in metastatic MCC (NCT04261855) that expresses somatostatin receptors in up to 77% of cases [106].

Oncolytic viruses are engineered to infect and replicate in tumor cells, causing lysis of tumor cells and thus stimulating the anti-tumoral immunity. Intralesional talimogene laherparepvec (T-VEC), an oncolytic recombinant herpes simplex type-1 virus-based agent, is currently being tested in combination with radiation therapy (NCT02819843) and nivolumab (NCT02978625).

The immune response is regulated by multiple pathways, both stimulatory and inhibitory. The latter include not only PD-1, PD-L1, and CTLA-4, but also lymphocyte activation gene-3 (LAG-3), an immune checkpoint protein that can cause immune exhaustion due to negative regulation of T-cell function [107,108]. The IgG4 human monoclonal antibody anti-LAG-3 relatlimab is currently under investigation in combination with nivolumab in a phase I/II trial (NCT02488759).

In metastatic MCC, the combination of tremelimumab, durvalumab, and a toll-like receptor 3 (TLR3) agonist PolyICLC is under evaluation in a trial phase I/II trial (NCT02643303). Activation of TLR3 can modulate the tumor microenvironment and potentiate the activity of other checkpoint inhibitors [86]. Other studies are investigating the use of the synthetic TLR4 agonist GLA–SE (NCT02035657) and of TTI-621, a recombinant fusion protein targeting CD47 (NCT02890368).

There are multiple ongoing clinical trials that are assessing the efficacy and safety of experimental immunotherapy approaches that target other pathways of the immune response. Among these, a phase II study (NCT02465957) is testing the combination of activated NK-92 natural killer (NK) cell infusions with ALT-803 (Interleukin-15) in patients with advanced MCC, getting advantage of the fact that activated NK cells could promote tumor cell lysis without the need for co-stimulatory molecules. In the Quilt-3.055 phase II trial, ALT-803 is also being tested in combination with PD-1/PD-L1 inhibitors for up to 16 cycles in patients with advanced solid tumors, including MCC or SCLC, progressed after an initial response on PD-1/PD-L1 inhibition (NCT03228667). A phase I trial is evaluating the injection of the IFx-Hu2.0 plasmid DNA construct into a target lesion, facilitating the localized expression of the highly immunogenic Emm55 protein on tumor cells (NCT04160065). Consequently, a cascade of immune events is activated towards both injected and non-injected lesions.

Chimeric antigen receptor T-cells (CAR–T) therapy is a rapidly evolving field among immune oncology treatments. CARs are genetically engineered receptors that enhance T-cell function, redirecting their specificity against select antigens [109]. First dramatic results were observed in hematologic malignancies, but new strategies are being developed in solid tumors as well [110]. In MCC, MCPyV antigens might be a promising target due to the important role of MCPyV in the genesis of MCC [81].

## 9. Conclusions

Immunotherapy is potentially a powerful weapon to help our NEN patients, but to date, the optimal strategy has not been identified yet. Future efforts should focus on finding the best way to include immunotherapy in the NEN treatment scenario, including the definition of the most appropriate setting, combination, and treatment sequence. There is solid evidence that the sooner the treatment, the better in immunotherapy, as also demonstrated by experience in MCC and SCLC [20,22,66], and the question is whether outcomes can be further improved through better patient selection or treatment combinations. It is likely that in poorly-differentiated NEC, the same considerations hold true, but joint efforts are needed to prove it in this rare population, with some promising trials already ongoing [64]. This can be due to either higher TMB or a higher expression of PD-L1 on tumor cells and a greater presence of TILs compared to well-differentiated NENs. For well-differentiated NETs, immunotherapy is still far from routine clinical application. The main limitations for the development of these strategies in NEN are the lack of predictive biomarkers, the rarity of the disease, and the long history of well-differentiated NEN that prevents OS from being used as a readily available endpoint. Future treatment approaches should include evaluation of synergistic combinations of different immune checkpoint inhibitors or the combination of immune checkpoint inhibition with other anticancer treatments, such as TKIs, especially those inhibiting VEGF, chemotherapy, and radiotherapy. Also, placing immunotherapy after chemotherapy could improve immune checkpoint inhibition efficacy by exploiting chemotherapy’s ability to increase TMB.

## Figures and Tables

**Table 1:** Studies discussed in the text and their main results divided by type of NEN. Abbreviations: Exp, experimental; mOS, median overall survival; mPFS, median progression-free survival; ORR, objective response rate; NEN, neuroendocrine neoplasia; HR, hazard ratio; CI, confidence interval; PD-L1, programmed cell death ligand 1.

NEN	Trial Name and Reference	Experimental Treatment/Control	Line of Therapy	Phase	OS	PFS	ORR
Small cell lung cancer	IMpower-133, 2019 [30]	Exp: atezolizumab + carboplatin/etoposide Control: carboplatin/etoposide	I line	III	mOS Exp: 12.3 months Control: 10.3 months (HR 0.70; 95% CI: 0.54–0.91)	mPFS Exp: 5.2 months Control: 4.3 months (HR 0.77; 95% CI: 0.62–0.96)	Exp: 60.2% Control: 64.4%
Small cell lung cancer	CASPIAN, 2019 [31]	Exp: durvalumab + carboplatin/etoposide Control: carboplatin/etoposide	I line	III	mOS Exp: 13.0 months Control: 10.3 months (HR 0.73; 95% CI: 0.59–0.91)	mPFS Exp: 5.1 months Control: 5.4 months (HR 0.78; 95% CI: 0.65–0.94)	Exp: 68% Control: 58% (HR 1.56; 95% CI: 1.10–2.22)
Small cell lung cancer	CA184-156, 2016 [54]	Exp: Ipilimumab + carboplatin/etoposide Control: carboplatin/etoposide	I line	III	mOS Exp: 11.0 months Control: 10.9 months (HR 0.94; 95% CI: 0.81–1.09)	mPFS Exp: 4.6 months Control: 4.4 months (HR 0.85; 95% CI: 0.75–0.97)	Exp: 58% Control: 58%
Small cell lung cancer	CheckMate-451, 2019 [55]	Exp: nivolumab Exp: nivolumab + ipilimumab Control: placebo	I line maintenance	III	mOS Exp: 9.2 months in nivolumab + ipilimumab arm Control: 9.6 months (HR 0.92; 95% CI: 0.75–1.12) Exp: 10.4 months in nivo arm Control: 9.6 months (HR 0.84; 95% CI: 0.69–1.02)	mPFS Exp: 1.7 months in nivolumab + ipilimumab arm Control: 1.4 months (HR 0.72; 95% CI: 0.60–0.87) Exp: 1.9 months in nivo arm Control: 1.4 months (HR 0.67; 95% CI: 0.56–0.81)	
Small cell lung cancer	CheckMate-331, 2018 [56]	Exp: nivolumab Control: topotecan/amrubicin	II line	III	mOS Exp: 7.5 months Control: 8.4 months (HR 0.86; 95% CI: 0.72–1.04)	mPFS Exp: 1.5 months Control: 3.8 months (HR 1.41; 95% CI: 1.18–1.69)	
Small cell lung cancer	CheckMate-032, 2016 [57]	Exp: nivolumab ± ipilimumab	≥II line	I/II	mOS nivolumab 3 mg/kg: 4.4 months nivolumab 1 mg/kg + ipilimumab 3 mg/kg: 7.7 months nivolumab 3 mg/kg + ipilimumab 1 mg/kg: 6 months	mPFS nivolumab 3 mg/kg: 1.4 months nivolumab 1 mg/kg + ipilimumab 3 mg/kg: 2.6 months nivolumab 3 mg/kg + ipilimumab 1 mg/kg: 1.4 months	nivolumab 3 mg/kg: 10% nivolumab 1 mg/kg + ipilimumab 3 mg/kg: 23% nivolumab 3 mg/kg + ipilimumab 1 mg/kg: 19%
Small cell lung cancer	KEYNOTE-158, 2018 [58] basket trial	Exp: pembrolizumab	≥II line	II	mOS 14.6 month in PDL1+ and 7.7 month in PDL1-	mPFS 2.1 month in PDL1+ and 1.9 month in PDL1-	35.7% in PDL1+ 6% in PDL1-
Small cell lung cancer	IFCT-1603, 2019 [59]	Exp: atezolizumab Control: chemotherapy	II line	II	mOS Exp: 9.5 months Control: 8.7 months (HR 0.84; 95% CI: 0.45–1.58)	mPFS Exp: 1.4 months Control: 4.3 months	Exp: 2.3% Control: 10%
Small cell lung cancer	MISP-MK3475, 2019 [60]	Exp: pembrolizumab + paclitaxel	II line	II	mOS: 9.1 months	mPFS: 5.0 months	23.1%
SCLC Low grade lung NEN Pancreatic NEC	KEYNOTE 028, 2019 [61]	Exp: pembrolizumab	≥II line	Ib	mOS: 9.7 months mOS: 21.1 months mOS:21.0 months	mPFS: 1.9 months mPFS: 5.7 months mPFS: 4.5 months	33% 12% 6%
Low grade GEP and lung NEN GEP NEC	CPDR001E2201, 2019 [62]	Exp: spartalizumab	≥II line	II			ORR overall 7.4% ORR in GEP NEC 4,8% ORR in thoracic NET 20%
NEN with Ki67 >10%	NCT03167853, 2020 [63]	Exp: toripalimab	≥II line	Ib	mOS: 9.1 months in PD-L1 ≥10% mOS: 7.2 months in PD-L1 <10% (HR 0.55; 95% CI: 0.24–1.23)	mPFS: 3.8 months in PD-L1 ≥10% mPFS: 2.2 months in PD-L1 <10% (HR 0.50; 95% CI: 0.24–1.06)	ORR was 42.9% (in PD-L1 expression ≥10%: 50.0%; in high TMB: 75.0%) ORR was 8.3% (in PD-L1 expression <10%)
NEN (no p-NEN)	DART/SWOG 1609, 2020 [64]	Exp: ipilimumab plus nivolumab	Any line (median II previous lines)	II	mOS: 11 months	mPFS: 4 months	25% (45% in high-grade and 0% in low-intermediete grade)
NET and NEC (any site)	NCT03074513, 2020 [65]	Exp: atezolizumab plus bevacizumab	≥II line	II		mPFS: 19.6 months in pNET mPFS: 14.9 months in extra-pNET	ORR: 20% in pNET ORR: 15% in extra-pNET
Merkel cell carcinoma	(CITN)09/KEYNOTE 017, 2019 [28]	Exp: pembrolizumab	I line	II		PFS rate at 6 months: 67%	56%
Merkel cell carcinoma	JAVELIN Merkel 200, 2018 [66]	Exp: avelumab	I line	II			62.1%
Merkel cell carcinoma	JAVELIN Merkel 200 2016 [29]	Exp: avelumab	≥II line	II	mOS: 12.9 months	1-year PFS: 30%	33%
Merkel cell carcinoma	CheckMate 358, 2017 [37]	Exp: nivolumab	I–III line	I/II	3-months OS rate: 92%	3-months OS rate: 82%	64%, I line: 71% II-III line: 63%

**Table 2:** Ongoing clinical trials of immunotherapy in NENs discussed in the text (source: clinicaltrials.gov; last accessed: 28 March 2020). Abbreviations: NEN, neuroendocrine neoplasia; N, sample size; PFS, progression-free survival; PFS-12, progression-free survival at 12 months; ORR, objective response rate; DFS, disease-free survival; DFS-12, disease-free survival at 12 months; RFS, relapse-free survival; CBR, clinical benefit rate; MTD, maximum tolerated dose; RP2D, recommended phase 2 dose.

Clinicaltrials.gov Identifier Name	N	Phase	Arm/Arms	Primary Outcome Measure	Estimated Primary Completion Date
**Lung NENs**					
NCT02554812 a Phase 1b/2 dose-optimization study to evaluate safety, pharmacokinetics, pharmacodynamics, and preliminary antitumor activity of avelumab (MSB0010718C) in combination with other cancer immunotherapies in patients with locally advanced or metastatic solid tumors.	620	Ib/II	Experimental: avelumab plus utomilumab avelumab plus PF-04518600 avelumab plus PD 0360324 avelumab plus utomilumab plus PF-04518600 avelumab plus CMP-001 Control: /	ORR	December 2022
NCT03126110 Phase 1/2 Study Exploring the Safety, Tolerability, and Efficacy of INCAGN01876 Combined With Immune Therapies in Advanced or Metastatic Malignancies	285	I/II	Experimental: Experimental: INCAGN01876 + nivolumab INCAGN01876 + ipilimumab INCAGN01876 + nivolumab + ipilimumab Control: /	ORR	January 2020
NCT03241173 A Phase 1/2 Study Exploring the Safety, Tolerability, and Efficacy of INCAGN01949 in Combination With Immune Therapies in Subjects With Advanced or Metastatic Malignancies	52	I/II	Experimental: Experimental: INCAGN01849 + nivolumab INCAGN01849 + ipilimumab INCAGN01849 + nivolumab + ipilimumab Control: /	ORR	November 2019
NCT03958045 Phase II Study of Combination Rucaparib With Nivolumab in Platinum-Sensitive Small Cell Lung Carcinoma Patients as Maintenance After Induction Therapy With Platinum Doublet	36	II	Experimental: rucaparib and nivolumab Control: /	PFS	July 2023
NCT03575793 A Phase I/II Study of Nivolumab, Ipilimumab, and Plinabulin in Patients With Recurrent Small Cell Lung Cancer: Big Ten Cancer Research Consortium.	55	I–II	Experimental: nivolumab, ipilimumab, and plinabulin Control: /	MTD PFS	September 2022
NCT03406715 Combination Immunotherapy–Ipilimumab–Nivolumab–Dendritic Cell p53 Vac—Patients With Small Cell Lung Cancer (SCLC)	41	II	Experimental: ipilimumab and nivolumab plus Dendritic Cell based p53 Vaccine (Ad.p53-DC) Control: /	DCR	April 2021
NCT04192682 Anlotinib Combined With Sintilimab as Second-Line Treatment or Beyond in Patients With Small Cell Lung Cancer	40	II	Experimental: anlotinib plus sintilimab Control: /	PFS	July 2021
NCT03728361 A phase II trial Nivolumab and Temozolomide in Treating Patients With Recurrent or Refractory Small-Cell Lung Cancer or Advanced Neuroendocrine Cancer	53	II	Experimental: nivolumab and temozolomide Control: /	ORR	December 2021
**Lung and GEP NENs**					
NCT03901378 A Phase II Trial of Pembrolizumab in Combination With Cisplatin or Carboplatin and Etoposide in Chemotherapy naïve Patients With Metastatic or Unresectable High-Grade Gastroenteropancreatic or Lung (Excluding Small Cell) Neuroendocrine Carcinoma	36	II	Experimental: pembrolizumab with carboplatin or cisplatin and etoposide Control: /	PFS	April 2021
NCT03591731 A GCO Trial Exploring the Efficacy and Safety of Nivolumab Monotherapy or Nivolumab Plus Ipilimumab in Pre-treated Patients With Advanced, Refractory Pulmonary or Gastroenteropancreatic Poorly Differentiated Neuroendocrine Tumors (NECs)	180	II	Experimental: nivolumab nivolumab + ipilimimab Control: /	ORR	September 2023
NCT04079712 A phase 2 study of XL184 (Cabozantinib) in combination with Nivolumab and Ipilimumab for the treatment of poorly differentiated neuroendocrine carcinomas	30	II	Experimental: nivolumab and ipilipimab and cabozantinib Control: /	ORR	October 2021
NCT03095274 Durvalumab (MEDI4736) Plus Tremelimumab for Advanced Neuroendocrine Neoplasms of Gastroenteropancreatic or Lung Origin (DUNE)	126	II	Experimental: durvalumab plus tremelimumab Control: /	CBR	April 2020
NCT03074513 A Phase II, Single-Arm Open-Label Study of the Combination of Atezolizumab and Bevacizumab in Rare Solid Tumors	160	II	Experimental: atezolizumab plus bevacizumab Control: /	ORR	March 2021
**Merkel cell carcinoma**					
NCT02196961 Prospective Randomized Trial of an Adjuvant Therapy of Completely Resected Merkel Cell Carcinoma (MCC) With Immune Checkpoint Blocking Antibodies (Nivolumab, Opdivo®; Ipilimumab (Yervoy®) Every 3 Weeks for 12 Weeks Vs. Observation	177	II	Experimental: nivolumab nivolumab plus radiotherapy Control: Observation	DFS-12	March 2022
NCT03271372 A Multicenter, Randomized, Double-Blinded, Placebo-Controlled, Phase 3 Trial of Adjuvant Avelumab (Anti-PDL-1 Antibody) in Merkel Cell Carcinoma Patients With Clinically Detected Lymph Node Metastases	100	III	Experimental: avelumab Control: Placebo	RFS	September 2024
NCT02584829 Localized Radiation Therapy or Recombinant Interferon Beta and Avelumab With or Without Cellular Adoptive Immunotherapy in Treating Patients With Metastatic Merkel Cell Carcinoma	8	I–II	Experimental: Avelumab plus radiotherapy plus recombinant interferon Beta Avelumab plus radiotherapy plus recombinant interferon Beta plus MCPyV TAg-specific Polyclonal Autologous CD8-positive T-Cells Control: /	Time to new metastasis	June 2022
NCT03071406 Randomized Study of Nivolumab+Ipilimumab+/- SBRT for Metastatic Merkel Cell Carcinoma	50	II	Experimental: nivolumab plus ipilimumab nivolumab plus ipilimumab plus SBRT Control: /	ORR	July 2023
NCT02819843 A Study of T-VEC (Talimogene Laherparepvec) With or Without Radiotherapy for Melanoma, Merkel Cell Carcinoma, or Other Solid Tumors	34	II	Experimental: Talimogene laherparepvec (TVEC) Talimogene laherparepvec (TVEC) plus hypofractionated Radiotherapy Control: /	ORR	June 2020
NCT02978625 Talimogene Laherparepvec and Nivolumab in Treating Patients With Refractory Lymphomas or Advanced or Refractory Non-melanoma Skin Cancers	68	II	Experimental: Talimogene laherparepvec plus nivolumab Control: /	ORR	January 2020
NCT02488759 An Investigational Immuno-therapy Study to Investigate the Safety and Effectiveness of Nivolumab, and Nivolumab Combination Therapy in Virus-associated Tumors (CheckMate358)	1100	I–II	Experimental: nivolumab nivolumab plus ipilimumab nivolumab plus relatlimab nivolumab plus daratumumab Control: /	Safety; ORR; Surgery delay	May 2022
NCT02643303 A Phase 1/2 Study of In Situ Vaccination With Tremelimumab and IV Durvalumab Plus PolyICLC in Subjects With Advanced, Measurable, Biopsy-Accessible Cancers	102	I–II	Experimental: IV durvalumab + IT/IM polyICLC IV durvalumab + IV tremelimumab + IT/IM polyICLC IV durvalumab + IT tremelimumab + IT/IM polyICLC Control: /	PFS-24	August 2022
NCT02035657 A Proof-of-Concept Trial of GLA—SE in Patients With Merkel Cell Carcinoma	10	I	Experimental: GLA-SE Control: /	Safety	March 2018
NCT02890368 Trial of Intratumoral Injections of TTI-621 in Subjects With Relapsed and Refractory Solid Tumors and Mycosis Fungoides	240	I	Experimental: TTI-621 TTI-621 + PD-1/PD-L1 Inhibitor TTI-621 + pegylated interferon-α2a TTI-621 + T-Vec TTI-621 + radiation Control: /	MTD/RP2D	December 2019
NCT02465957 QUILT-3.009: Patients With Stage III (IIIB) or Stage (IV) Merkel Cell Carcinoma (MCC)	24	II	Experimental: aNK (NK-92) Control: /	PFS	April 2019
NCT04291885 Immunotherapy Merkel Adjuvant Trial (I-MAT)	132	II	Experimental: avelumab Control: Placebo	RFS	December 2028
NCT03798639 Nivolumab and Radiation Therapy or Ipilimumab as Adjuvant Therapy in Treating Patients With Merkel Cell Cancer	43	I	Experimental: nivolumab plus radiation therapy nivolumab plus ipilimumab Control: /	% completing 12 months of treatment	31 December 2021
NCT03988647 Palliative RT and Anti-PD-1/PD-L1 Checkpoint Blockade in Metastatic Merkel Cell Carcinoma	30	II	Experimental: pembrolizumab plus radiation therapy Control: /	ORR	June 2026
NCT03304639 Pembrolizumab With or Without Stereotactic Body Radiation Therapy in Treating Patients With Advanced or Metastatic Merkel Cell Cancer	100	II	Experimental: pembrolizumab plus radiation therapy pembrolizumab Control: /	PFS	7 February 2022
NCT04160065 Immunotherapy With IFx-Hu2.0 Vaccine for Advanced MCC or cSCC	20	I	Experimental: IFx-Hu2.0 Control: /	Safety	September 2021
NCT03712605 STAMP: Surgically Treated Adjuvant Merkel Cell Carcinoma With Pembrolizumab, a Phase III Trial	500	III	Experimental: pembrolizumab +/- radiotherapy Control: Observation +/- radiotherapy	RFS, OS	31 October 2023
NCT04261855 Targeted Therapy and Avelumab in Merkel Cell Carcinoma (GoTHAM)	65	I/ II	Experimental: avelumab plus external beam radiation therapy avelumab plus Lutetium-177 (177Lu)-DOTATATE Control: /	PFS-12	January 2024
NCT03589339 NBTXR3 Activated by Radiotherapy for Patients With Advanced Cancers Treated With An Anti-PD-1 Therapy	60	I	Experimental: NBTXR3 Control: /	RP2D	30 March 2023
**Merkel cell carcinoma and SCLC**					
NCT04272034 Safety, Tolerability, Pharmacokinetics, and Pharmacodynamics of INCB099318 in Participants With Advanced Solid Tumors	140	I	Experimental: INCB099318 Control: /	Safety	30 October 2023
NCT03841110 FT500 as Monotherapy and in Combination With Immune Checkpoint Inhibitors in Subjects With Advanced Solid Tumors	76	I	Experimental: FT500 FT500 plus immune checkpoint inhibitors Control: /	Safety	June 2022
